# “Back Rx, a personalized mobile phone application for discogenic chronic low back pain: a prospective pilot study”

**DOI:** 10.1186/s12891-022-05883-9

**Published:** 2022-10-19

**Authors:** Vijay B. Vad, Antonio Madrazo-Ibarra, Deborah Estrin, John P. Pollak, Kaitlin M. Carroll, Deneen Vojta, Amoli Vad, Camilla Trapness

**Affiliations:** 1 Assistant Professor, Hospital for Special Surgery, New York, NY USA; 2 Hospital for Special Surgery, Research Fellow, New York, NY USA; 3 Research Fellow, Department of Physiatry, Hospital for Special Surgery, 519 E 72nd St, New York, NY 10021 USA; 4 Associate Dean of Computer Science, Cornell Tech, New York, NY USA; 5 Cornell Tech, Senior Research-in-Residence, New York, NY USA; 6 Hospital for Special Surgery, Research Assistant, New York, NY USA; 7 Executive Vice President, UnitedHealth Group Research & Development, Minnetonka, MN USA; 8 Hospital for Special Surgery, Research Coordinator, New York, NY USA

**Keywords:** Low back pain, Chronic low back pain, Exercise therapy, Telemedicine, Mobile applications, Self-management

## Abstract

**Background:**

Intervertebral disc pathology is the most common identifiable cause of chronic lower back pain (CLBP). There are limited conservative alternatives to treat discogenic axial CLBP. Back Rx is a mobile application (app) developed to treat patients with this condition, following the Back Rx exercise program, assisted by a virtual coach.

**Methods:**

Patients 18 to 65 years of age, with axial CLBP (more than 3 months), and evidence of lumbar disc pathology by magnetic resonance imaging (MRI) were enrolled to the study. Patients’ symptomatology was prospectively evaluated at baseline and after 3 months of using the Back Rx app. The main outcome of the study was back pain evaluated using the visual analog scale (VAS) for pain. Secondary outcomes were the patient's functionality, the weekly pain medication intake, the patients’ adherence to the app, and the patients´ satisfaction rate.

**Results:**

Seventy-five patients with CLBP were enrolled in the study. All patients had a statistically significant improvement from baseline to final follow-up in the average VAS scores, and the functionality evaluations. Average VAS scores decreased from 5.17 ± 2.1 at baseline to 3.8 ± 2.6 at final follow-up (*P* = 0.016). Patients showed a significant decrease in the number of pain medications taken during a week (*P* = 0.001). Overall compliance with the app was 52%, and 65% of the patients rated the overall experience as good or excellent.

**Conclusion:**

The Back Rx app decreased pain and increased function in patients with discogenic axial CLBP compared to their baseline status. Further measures are needed to increase patients' compliance with the app and the Back Rx program.

**Trial registration:**

Retrospectively registered in 2/2/2017 NCT03040310 (ClinicalTrials.gov).

**Supplementary Information:**

The online version contains supplementary material available at 10.1186/s12891-022-05883-9.

## Background

Chronic low back pain (CLBP), defined as low back pain for > 3 months, is acknowledged as the most common cause of disability worldwide [1, 2]. Patients with CLBP suffer from high direct costs related to medical care and indirect costs related to loss of productivity [3]. These costs, in addition to the pain and disability related to the disease, greatly impact the patient’s quality of life and mental health [3]. While more than 85% of low back pain cases have no specific underlying cause, when identified, they are most commonly caused by intervertebral disc pathologies [4, 5]. Intervertebral disc pathologies can cause axial symptoms such as back pain or peripheral symptoms like nerve irritation and radiculopathies. There are many conservative treatments for peripheral discogenic CLBP, however, there are limited conservative alternatives for axial symptoms [6]. An effective non-invasive option for axial CLBP may improve patients’ symptoms, avoid unnecessary risks, reduce major costs, and prevent adverse effects related to the long-term use of pain medications.

Current treatment guidelines recommend starting with conservative measures to treat patients with CLBP [7]. While self-management (including educating patients about their disease and encouraging them to continue with their daily activities) remains the main pilar of the conservative treatment, exercise is recommended as one of the first treatment measures [7]. Physical exercise has demonstrated to reduce pain and improve function in patients with CLBP [8, 9]. But exercise programs need a higher patient adherence to obtain positive results [10, 11]. Mobile health (mHealth), or mobile phone applications (apps) have increased patients’ engagement to exercise programs obtaining higher adherence compared to regular self-managed programs [12–17]. Although the number of health apps available for back pain has greatly increased, none of them were developed specifically for axial discogenic CLBP.

Back Rx app was created to treat discogenic axial CLBP following the Back Rx rehabilitation program. This program was developed in 2004 and has showed great success in preventing and treating patients suffering from this condition [18, 19]. It consists of a 15-min exercise program that combines yoga and Pilates exercises promoting strength, coordination, and stability. The Back Rx app was released to increase patients’ adherence to the program by delivering personalized coaching support, and most importantly addressing the symptomatology of patients. But, there is no study evaluating the effectiveness of the Back Rx app in delivering the exercise program to patients, and improving pain and function. Therefore we decided to conduct a pilot study using the Back Rx app in patients with discogenic axial CLBP, evaluating clinical outcomes and patients’ adherence to the program, laying the basis for future studies.

## Materials and methods

### Study design

This prospective clinical trial was conducted at a single institution (NCT03040310, retrospectively registered on 2/2/2017 at ClinicalTrials.gov). Patients from a Physical Medicine and Rehabilitation private practice (affiliated to the Hospital for Special Surgery) were enrolled if they had all the following inclusion criteria: 18 to 65 years of age, axial symptoms of CLBP (> 3 months of duration), low back pain exacerbated by sitting or with lumbar flexion, evidence of lumbar intervertebral disc pathology (including bulging, protrusion, extrusion, or annular fissures) on magnetic resonance imaging (MRI), computer literate, and owned a smartphone (iPhone models 5S and later or Android models 2.3 or later). Exclusion criteria included: concurrent pathology that may contribute to the patient’s axial low back symptoms (e.g., spondylolysis, spondylolisthesis, facet arthropathy), severe lumbar disc degeneration before beginning the Back Rx exercise program, any peripheral neurological symptom attributed to the intervertebral disc pathology, history of lumbar spine surgery, or history of previous spine trauma. Patients were followed-up for 3 months after starting to use the Back Rx app. The last patient evaluation was performed in September 2016.

The study was performed according to Good Clinical Practice guidelines and principles of the Declaration of Helsinki [20, 21]. It was approved by the local ethics committee and the internal review board of the Hospital for Special Surgery (New York, NY). Written informed consent was obtained from all patients.

### Intervention

**Fig. 1:**
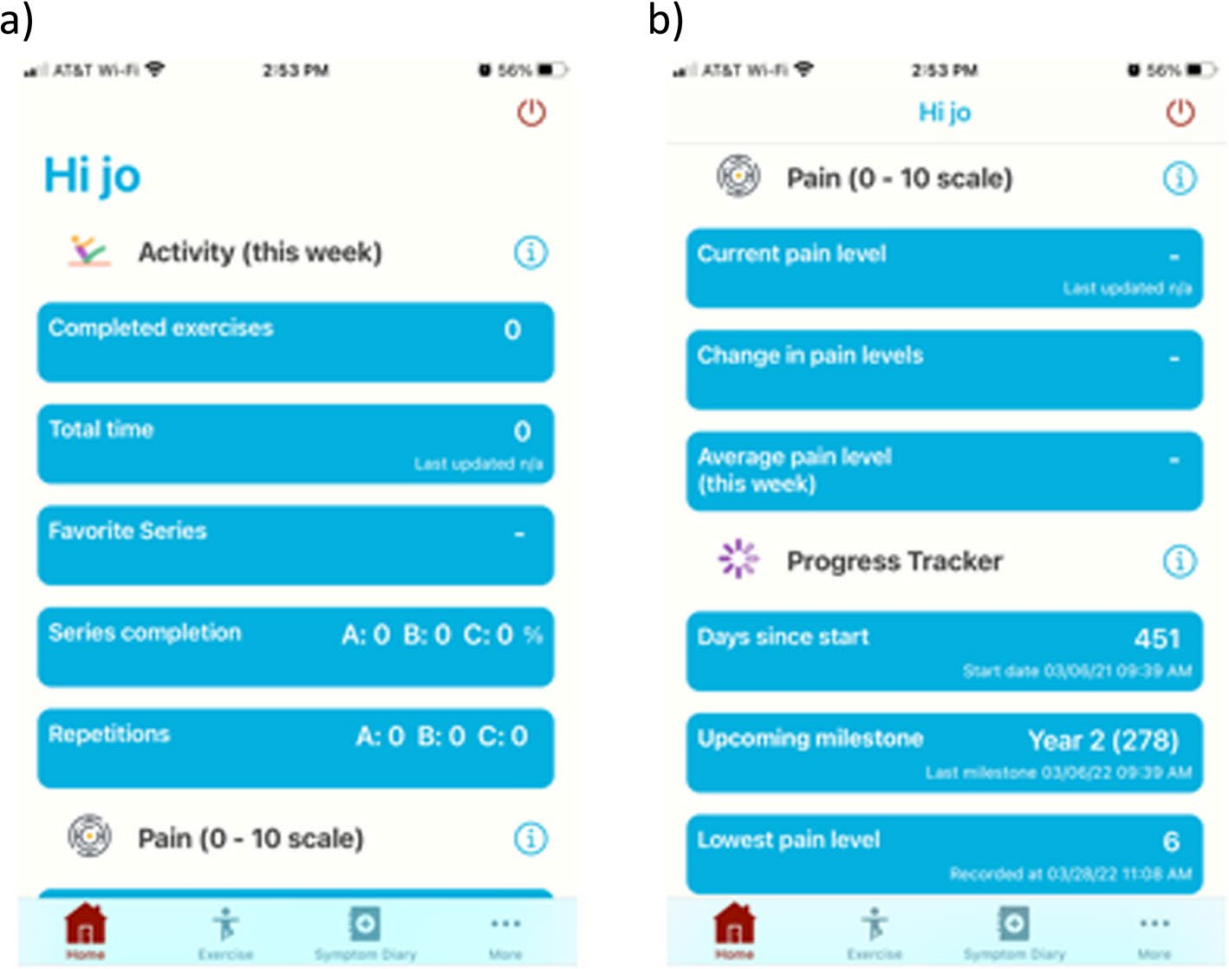
Screenshots of the mobile phone application. **a** Shows the home screen (**b**) shows the pain and progress tracker. Here patients can freely modify their current pain as it changes during the exercise program

Eligible patients were enrolled in a mHealth-based 3-month physical exercise program. Physical exercise has been consistently recommended as one of the first treatment options for patients with CLBP [7]. Patients received a mobile phone app (“Back Rx”) free of charge to monitor and manage their low back pain (Fig. 1). Before starting the 3-month program, patients were given a detailed educational course on the app and all their questions were answered. The exercise program included self-directed rehabilitation video tutorials based on the Back Rx program, a virtual coach that sent daily messages to the patients reminding them to perform the exercises, and several motivational messages based on the patient’s daily, weekly and monthly performance. Patients were instructed to perform the exercises for 15 min every day, following the instructional videos that encouraged them to perform the exercises correctly. A detailed description of the exercises can be found in the supplementary files.

Patients were instructed to continue their regular pain medications as needed including non-steroidal anti-inflammatory drugs (NSAIDS), dietary supplements, and opiate derivates.

### Outcomes

The main outcome of the study was back pain evaluated using the visual analog scale (VAS) for pain. Secondary outcomes were the patient's functionality, the weekly pain medication intake, the patients’ adherence to the app, and the patients´satisfaction rate.

### Evaluation

We evaluated patients’ pain using the VAS for back pain (scores range from 0–10, the higher score indicating worst pain) [22]. Patients functionality was evaluated using Your Activities of Daily Living (YADL) (0–100%, the higher the percentage the more symptomatic and less functional) [23], and the Oswestry Disability Index (ODI) for function (0–100, the higher the score the greater the disability) [24]. All patient reported outcomes were answered through the app at 3 weeks, 6 weeks, and 3 months after initiating the Back Rx program. The weekly pain medication intake was assessed with the medication for daily living questionnaire which patients answered daily at the end of each day.

YADL is an image-based survey that evaluates a patient’s functional status using images of everyday activities taken from the Western Ontario and McMaster Universities Arthritis Index and Boston Activity Measure for Post-Acute Care [25, 26]. Patients selected images of activities that caused them back pain. Once an activity was selected, the patients graded the activity as “easy”, “moderate”, or “hard” depending on the difficulty they had performing the activity. The difficulty was given a score of 0 = easy, 0.5 = moderate, and 1 = hard. The full assessment included 47 images. At the end of the assessment, all scores were added, divided by the total number of activities selected as pain triggers, and multiplied by 100 to obtain a percentage.

Medications of Daily Living is a visual app log used to evaluate “what” and “how many” medications the patient ingested in the last 24 h. A previous study explains both YADL and Medications of Daily Living in more detail [23].

### Patient compliance and satisfaction

Patients were required to perform the exercises at least three times per week to be considered “active”. At the end of each instructional video, patients confirmed they had watched the whole video and performed the exercises by answering some questions. If patients remained active for the whole 12 weeks, they were considered “compliant”. A patient satisfaction questionnaire was performed at the end of the program.

### Adverse events

Adverse events related to using the Back Rx app were evaluated throughout the whole follow-up. Patients were instructed to record adverse events they experienced while following the exercise program. In the case a patient presented a severe adverse event (life threatening) or an adverse event that was limiting their daily life, they were instructed to contact the principal investigator right away and stop using the Back Rx app.

### Statistical analyses

All continuous data with a normal distribution were expressed in terms of mean ± SD; the categorical data were expressed as frequencies and percentages. Data with non-normal distribution were expressed in terms of median and variance. Shapiro–Wilk test was performed to assess the normality of continuous variables. All data with normal distribution were compared using the Student t-test for continuous variables and chi-square for categorical variables. Nonparametric tests were used to compare data with non-normal distribution. Pearson's correlation analysis was performed, *P* values < 0.05 were considered statistically significant. Statistical Analysis was performed using SPSS software version 26 (IBM).

## Results

**Fig. 2:**
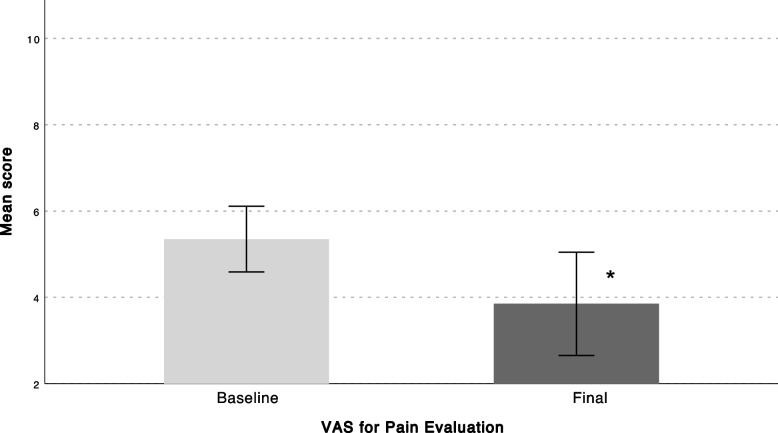
Mean (95% CI); VAS = Visual Analog Scale, * = *P* < 0.05

**Fig. 3:**
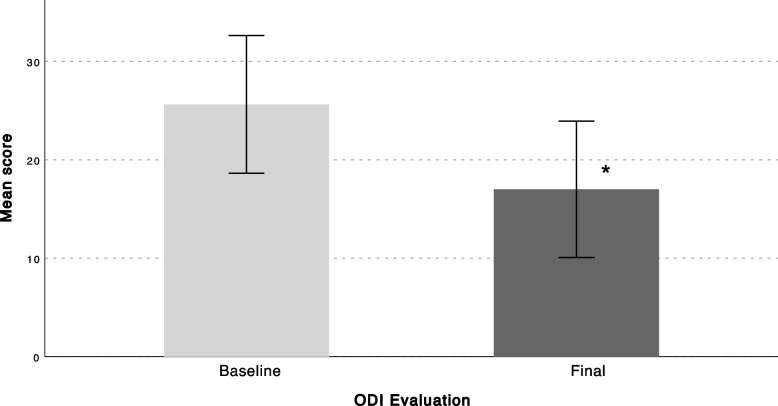
Mean (95% CI); ODI = Oswestry Disability Index * = *P* < 0.05

**Fig. 4:**
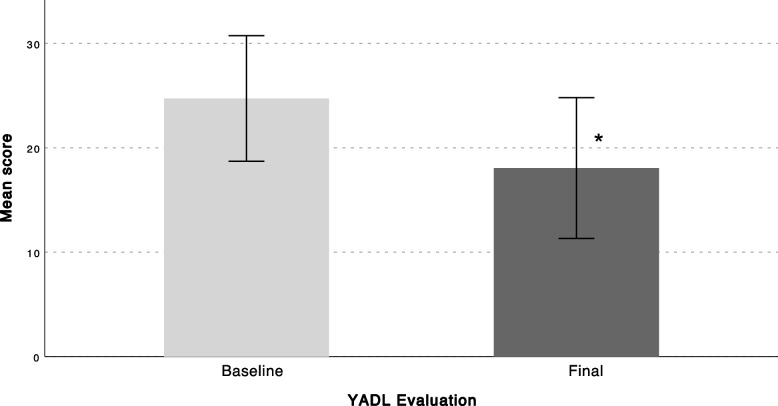
Mean (95% CI); YADL = Your Activities of Daily Living * = *P* < 0.05

**Fig. 5:**
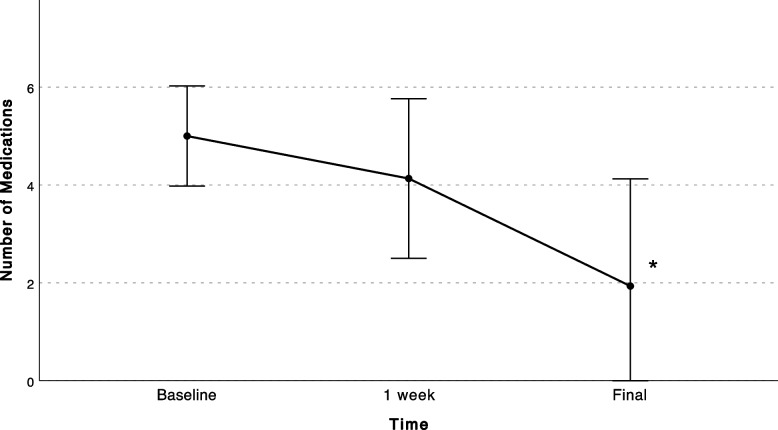
Average Pain Medications Taken in a Week. Mean (95% CI); Final = 3 months. * = *P* < 0.05 Baseline versus Final

A total of 75 patients with axial discogenic CLBP were enrolled in the study, 11 of which dropped out due to technical problems using the app. Twenty-five patients discontinued using the app before finishing the 3-moth program. A total of 39 patients completed the program and were included in the final analysis. Patients who discontinued using the app had statistically significantly lower body mass index (BMI) compared to patients who completed the follow-up (Table 1). All PROMs showed a significant decrease from baseline to final follow-up (Figs. 2, 3 and 4). Average VAS scores decreased from 5.17 ± 2.1 at baseline to 3.8 ± 2.6 at final follow-up (*P* = 0.016) (Fig. 2), ODI scores from 25.7 ± 13.8 to 18.2 ± 13.6 (*P* = 0.001)(Fig. 3), and YADL scores from 23.9 ± 12.2 to 18.1 ± 12.2 (*P* = 0.009) (Fig. 4). Patients showed a decrease in the average pain medications taken each week since the first week of the program and reached a statistically significant decrease at 3 months compared to baseline (*P* = 0.001) (Fig. 5). A weak positive correlation was observed between YADL and ODI using full assessment YADL and baseline ODI scores (*R*2 = 0.2542, *P* = 0.0062). Overall adherence with the app was 52% (39/75), with 65% (25/39) of the patients rating the overall experience with the program as good or excellent. No adverse events were reported throughout the follow-up.

## Discussion

This prospective pilot study evaluating Back Rx as a mHealth instrument for patients with discogenic CLBP showed patients appeared to have significantly less back pain after 3 months of using the app, compared to their baseline pain, according to the VAS for back pain. Patients' functionality significantly improved, and the average amount of pain medications taken daily decreased to half of the baseline medications taken daily. Patients went from having a “moderate disability” according to the average ODI at baseline, to “minimal disability” after 3 months of using the Back Rx app [27]. Overall, 52% of patients adhered to the program, and more than half of the patients (65%) affirmed to have a good or excellent experience with the app. These promising findings suggest the Back Rx app could be a potential tool for managing axial discogenic CLBP.

Studies evaluating exercise programs for CLBP have suggested exercise is effective in reducing and preventing pain, as well as improving function [8, 9]. A recent Cochrane systematic review by Hayden et al. [8] reported exercise is more effective than no treatment, or common treatments for CLBP in the short and medium-term. However, due to the heterogeneous studies and clinical findings, the effect of exercise programs on CLBP may be downsized. In accordance with what they reported, we observed that after following the Back Rx program, patients with CLBP show improved pain and function after 3 months. As described by Gordon et al. [28] exercise increases core muscular strength providing support to the lumbar spine, improves flexibility resulting in a greater range of motion and correcting the posture; and increases the blood flow to the soft tissues in the back. In addition to these physical changes, exercise may have a psychological effect on improving mood and function in patients [14]. The Back Rx program combines both Yoga and Pilates which could address both this physical and mental wellness causing patients with CLBP to feel better [29, 30].

As mentioned earlier, several mobile apps are being developed addressing chronic musculoskeletal pain, with most apps offering guided exercise programs. [14, 17, 31–33] All available apps focusing on CLBP have showed to decrease pain and increase function, similar to the findings with the Back Rx app [31–35]. However, there is a controversy regarding if apps are better than conventional self-managed exercise programs. On the one hand, some studies indicate that apps generate greater pain and function improvement in patients with CLBP [31]. On the other hand, Amorim et al. [33] and Chhabra et al.[32] observed both patients using an app and patients following conventional treatment had similar pain outcomes. Future randomized controlled trials are necessary to determine the effectiveness. In theory, mobile phone apps should enhance exercise programs by guiding the patient into performing the exercises adequately and motivating patients, therefore generating better outcomes, however more randomized trials are needed to come up with conclusions regarding the superiority of one over the other.

Patients’ adherence to exercise programs determines how successful the outcomes are, with patients adhering more to the programs usually obtaining better results [14]. Although mobile apps were developed trying to improve the adherence to exercise programs, a recent systematic review by Lewkowicz et al. reported studies evaluating mobile apps for managing low back pain have dropout rates of 2%-82% [35]. Lower dropout rates were observed in studies promoting support interventions such as personalized messages, push notifications, and activity recommendations [35]. Our study showed that 52% of patients adhered to the Back Rx program, and 48% of patients discontinued using the app, congruent to what is previously described. Despite the brief duration of the exercises performed daily in the Back Rx program (15 min) and the daily messages sent by the virtual coach motivating and reminding patients to do their exercises, as suggested by Beinart et al. [36], our dropout rate remains higher than what would be desired. Oakley-Girvan et al. [37] analyzed what measures worked best to maintained patients engaged to mobile app interventions and some of these measures included app personalization, prompts to use the app, connecting with other patients using the app, setting short term goals, and increasing the interaction with the doctor or researcher. By implementing these measures, we could potentially increase patient’s adherence to the Back Rx app, and therefore enhance the benefits from following the program.

Our study results show that the Back Rx app can potentially be an effective mHealth tool delivering the Back Rx program to patients with discogenic CLBP and axial symptoms but needs further improvements. Being a more affordable alternative than surgical or minimally invasive procedures, the Back Rx app could help reduce costs related to health care use and productivity loss, as well as avoiding unnecessary risks related to more invasive procedures [38]. By decreasing pain, and the medications needed daily to alleviate symptoms, the risk of narcotic addiction greatly decreases. This finding may be crucial due to the narcotic crisis we are currently facing, where almost ¾ of opiate-derived abusers started by taking legally prescribed medications [39].

A number of limitations need to be noted regarding the present study. First of all, there was no control group to compare with, making it difficult to evaluate the effectiveness of the app. Therefore, a future placebo-controlled or randomized clinical trial is needed. A second limitation is that only 52% of the patients enrolled were analyzed at the final follow-up. This can be due to could be possibly due to a selection bias. Eleven patients of patients dropped out due to technical issues, and 25 patients did not finish the exercise program. However, the latter 25 patients had a lower baseline BMI and may have showed positive outcomes early in the program explaining why they decided to suspend using the app [40]. As suggested by Ross et al. [17] patients that manage their pain better have less compliance to exercise apps. Another important limitation to mention is the short follow-up of 3 months. CLBP is a fluctuating pathology with patients´ pain improving but recurring afterwards, suggesting a longer follow-up could be necessary. Finally, this study was performed in a single institution and a single private practice which could have biased the results and decreased the response variability. Further pragmatic multicenter studies may give a better understanding of the real-world scenario of patients with discogenic CLBP.

## Conclusion

In conclusion, the Back Rx app showed improvements related to decrease pain and increase function in patients with discogenic axial CLBP compared to their baseline status. Further measures are needed to increase patients' adherence with the app and the Back Rx program. This app may be an effective conservative alternative to managing patients with CLBP, offering no risks or major economic implications.

**Table 1:** Demographic Characteristics of Patients

Characteristic	Finished Follow-up (*n* = 39)	Discontinued App use (*n* = 25)	*P*-value
Sex, n (%)			
Female	25 (64)	13 (52)	0.925
Male	14 (36)	12 (48)	
Age, (years)	44.4 ± 15.6	45.0 ± 14.7	0.893
BMI kg/m^2^	25.3 ± 4.0	23.0 ± 2.5	0.024

Data presented as mean ± standard deviation unless otherwise specified

*BMI* Body mass index

## Supplementary Information

**Additional file 1.** The back rx exercises. The Back Rx program was developed to increase flexibility, strength, and endurance with the objective of improving low back pain. It combines physical therapy and rehabilitation, yoga, and Pilates, taking the benefits of all three.

## Data Availability

The datasets generated and/or analyzed during the current study are not publicly available due to patients’ confidentiality but a coded copy of the dataset is available to all public upon request to the corresponding author.

## Abbreviations

CLBP: Chronic low back pain
app: Application
MRI: Magnetic Resonance Imaging
PROMs: Patient-Reported Outcome Measures
VAS: Visual Analog Scale
ODI: Oswestry Disability Index
YADL: Your Activities of Daily Living
mHealth: Mobile health
