# Supplementary material for: “Back Rx, a personalized mobile phone application for discogenic chronic low back pain: a prospective pilot study”
Source: BMC Musculoskelet Disord. 2022 Oct 19;23:923. doi: 10.1186/s12891-022-05883-9 (PMC9580128; doi:10.1186/s12891-022-05883-9)
Supplement: Supplementary file 1 — Additional file 1. The back rx exercises. The Back Rx program was developed to increase flexibility, strength, and endurance with the objective of improving low back pain. It combines physical therapy and rehabilitation, yoga, and Pilates, taking the benefits of all three. [file 12891_2022_5883_MOESM1_ESM.docx]

**The Back Rx Exercises**

The Back Rx program was developed to increase flexibility, strength, and endurance with the objective of improving low back pain. It combines physical therapy and rehabilitation, yoga, and Pilates, taking the benefits of all three.

It comprises a series of exercises with the following breakout:

| Physical Therapy/ Rehabilitation | Yoga | Pilates |
| --- | --- | --- |
| 50% | 30% | 20% |

This series emphasizes on isometric muscle work from physical therapy, increasing core muscle flexibility and preparing the body for the increased stress of strength and endurance training.

Both yoga and Pilates exercises have been modified to avoid positions and movements that can injure a weak back.

Each series takes around 15 minutes to complete and we recommend patients do the exercises at least 3 times a week.

**Exercises.**

Hold each posture for five full deep breaths.

1. Sun salutation lying down (flat on your back)
   1. Inhale and lift your hands above your head.
   2. Exhale and bring your hands back to your sides


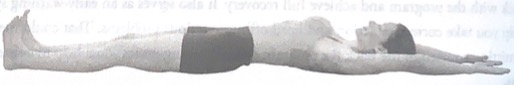


1. Bridging
   1. Lying on your back and with your knees bent, lift your hip of the floor and maintain that position


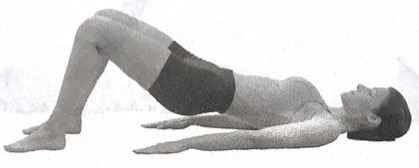


1. Abdominal crunch
   1. Lying on your back, with your knees bent and your hands by your side (flat on the floor), raise your shoulders and head while squeezing your abdominals.
   2. Maintain your head off the floor for 5 breaths and then come back to resting position


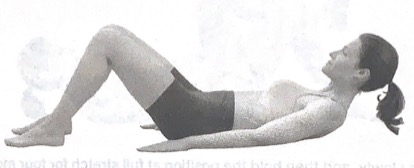


1. Knee to chest
   1. Lying on your back and with your knees bent
   2. Clasp your hands in the crook of one bent, pull the knee towards your chest and lift your head off the floor.
   3. Maintain that position for 5 breaths and then come back to resting position
   4. Repeat the exercise with the other knee


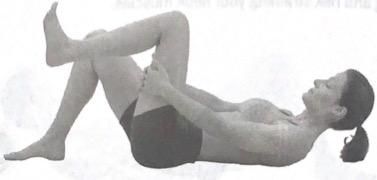


1. Abdominal crunch with leg flexed
   1. Lie flat on your back with one knee bent and keeping the other leg straight.
   2. Lift your shoulders and head off the floor and maintain that position for 5 deep breaths
   3. Come back to resting position
   4. Repeat exercise with the other knee bent


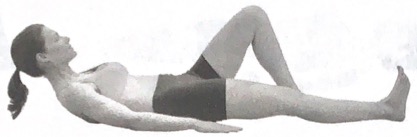


1. Tree pose
   1. Lying flat on your back with your knees straight, bend one knee, place the sole of that foot on the inside of your other knee and let the bent knee fall to the side.
   2. Hold that position for 5 deep breaths and then come back to resting position
   3. Repeat exercise with the other leg


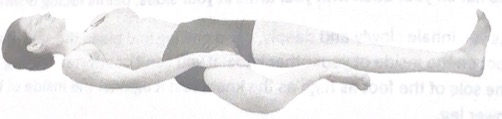


1. Bound angle posture
   1. Lying flat on your back with your knees straight, bring both of your feet toward your groin, so that your heels touch but your toes do not.
   2. Let your knees fall to the side and maintain that position for 5 deep breaths


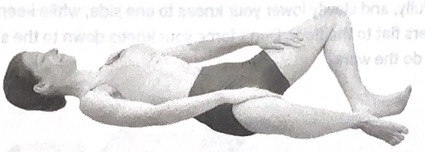


1. Lumbar rotation double knee
   1. Lying on your back with your knees bent, extend your arms straight out of your shoulders with your palms facing down
   2. Slowly lower your knees to one side, while keeping your shoulders flat to the floor.
   3. Hold that position for 5 deep breaths
   4. Repeat the exercise lowering your knees to the other side


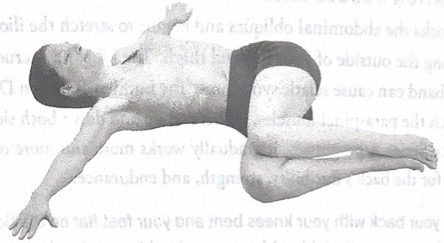


1. Lumbar rotation single knee
   1. Lie on one side supporting your head on one hand.
   2. Bend your top leg and cross it over the bottom so that your bent knee touches the floor and the foot rests on the floor in front of the bottom knee.
   3. Maintain this position
   4. Come back to resting position and repeat exercise facing the other way


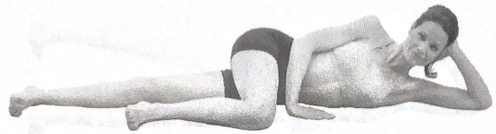


1. Hip hikers
   1. Lie on your side with one hand holding up your head and the other placed in front of your chest
   2. Keep both legs straight and slowly raise one leg
   3. Hold that position for 5 breathes
   4. Come back to resting position
   5. Repeat exercise on the other side


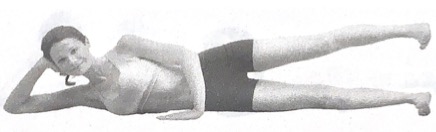


1. Staff posture
   1. Sit on the floor with your legs straight, toes pointing to the ceiling and your hand positioned slightly behind your hip with your palms flat on the floor
   2. Maintain your back and neck as straight as possible
   3. Hold that position


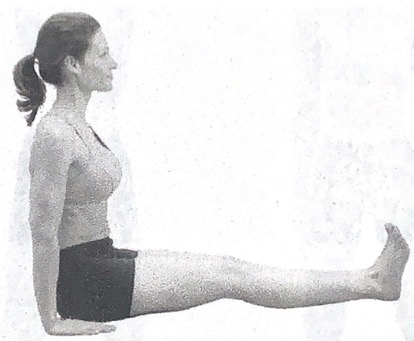


1. Sun salutation on knees
   1. Carefully move to a kneeling position with your shoulders, hip and thighs in line
   2. Inhale and sweep your arms straight up at the sides, lengthening your back and neck as much as possible
   3. Exhale slowly and bring your arms back down to your sides


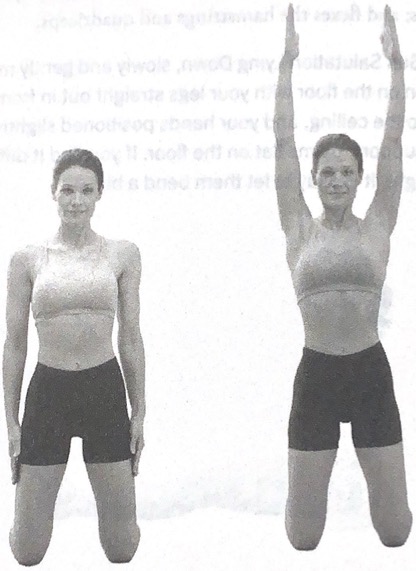


1. Locust posture
   1. Lie on your stomach with your arms straight at your sides
   2. With your legs completely straight, raise one leg and keep it off the floor for 5 deep breaths
   3. Repeat exercise with opposite leg


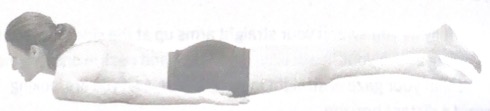


1. Back extension
   1. Take a position on all fours
   2. Carefully lift one leg and extend it backwards as you inhale
   3. Maintain that position while lengthening the back and neck
   4. Return to starting position
   5. Repeat exercise with opposite leg


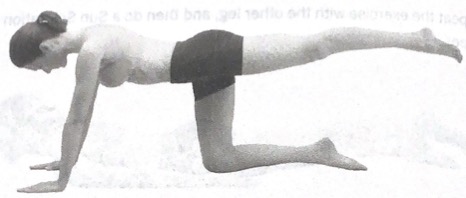


1. Cat stretch
   1. Take a position in all fours
   2. Inhale and arch your back
   3. Hold the arch position for 5 deep breaths
   4. Then let gravity pull your belly button toward the floor and hold that position


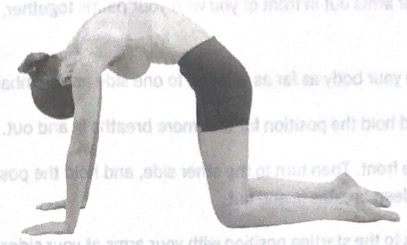


1. Flexibility prayer
   1. In a standing position with your feet the same width apart as your shoulders, and your hands by your side, lift your arms straight in front of you and bring your palms together
   2. Slowly turn your body as far as you can to one side
   3. Hold that position for 5 deep breaths
   4. Return to starting position
   5. Repeat exercise turning the opposite side


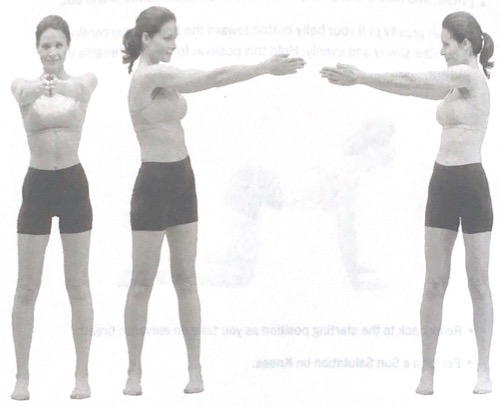


1. Sun salutation standing
   1. In a standing position with your feet the same width apart as your shoulders, and your hands by your side, inhale and sweep your arms up to the sides and above your head
   2. Exhale and bring your arms back down
   3. Repeat 5 times


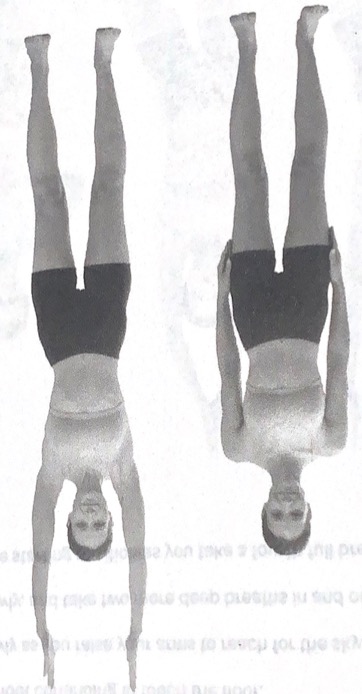


1. Standing tree pose
   1. In a standing position with your feet the same width apart as your shoulders, and your hands by your side, bring the heel of one foot to rest on the ankle of the other leg
   2. Inhale slowly as you raise your arms above your head
   3. Hold that position for 5 deep breaths
   4. Return to the starting position
   5. Repeat exercise with the opposite heel


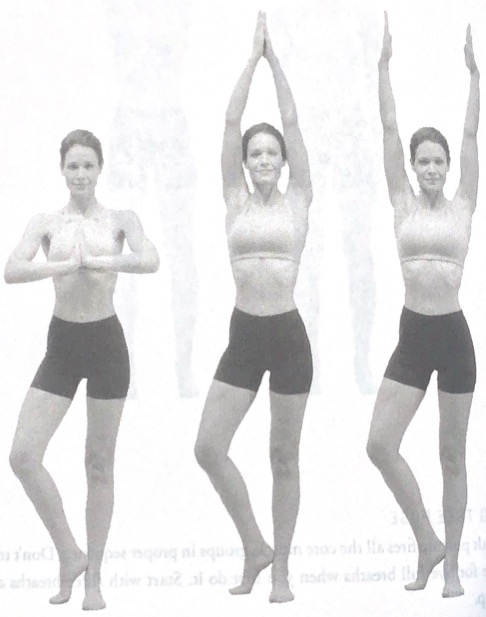


*All pictures and exercises were taken from the book *Back Rx.* 2^nd^ edition, with prior authorization from Dr. Vijay B. Vad

**Reference**

1. Vad V, Occhiogrosso P. Back Rx. 2nd ed. Penguin Random House LLC; 2019.
